# Supplementary material for: The individuality of single-frame functional brain connectivity
Source: Imaging Neurosci (Camb). 2026 Jun 17;4:IMAG.a.1254. doi: 10.1162/IMAG.a.1254 (PMC13277782; doi:10.1162/IMAG.a.1254)
Supplement: Supplementary Material [file IMAG.a.1254_supp.pdf]

Supplementary Material

tSNR and ReHo Calculation and Linear Mixed Effects Models

Temporal signal-to-noise ratio (tSNR) was calculated for each node by dividing the mean signal from the pre-band-pass filtered image node by the standard deviation of the signal from the final time series (post-band-pass filtering and motion correction). This was done to approximate the signal to noise ratio after removal of nuisance signal (i.e., low and high frequency signal, motion artifacts). The mean could not be directly taken from the final time series because the bandpass filtering approach mean-centered these data to zero, and the tSNR of each node was therefore also 0. Another common approach for calculating tSNR is to use the mean and standard deviation of the signal that has undergone minimal preprocessing. We replicated all findings with tSNR in this work using this second approach and found that results with this approach had no meaningful difference in interpretation (i.e., the relationships with ReHo and identification accuracy was unchanged), though tSNR values were lower than in the first approach described.

For each atlas parcellation of each scan, the mean (across nodes) tSNR was calculated and used for subsequent analyses. Regional homogeneity (ReHo) was calculated as Kendall's coefficient of concordance for the time series of the voxels within each atlas node. Therefore, each node had a ReHo value. For each atlas parcellation of each scan, the mean (across nodes) ReHo was calculated and used for subsequent analyses.

In Section 3.4, the average nodal tSNR and ReHo value for each scan were included in linear mixed effects models to determine whether identification accuracy was associated with average tSNR or ReHo. In the first analyses (in which finer resolution atlases featured more total nodes per scan), a linear mixed effects model was fit for each study. In each model, each

combination of scan and atlas parcellation was included as an observation, with the number of correctly identified volumes as the dependent variable for the corresponding observation. The average tSNR and ReHo values for the corresponding scan/atlas parcellation combination were included as fixed effects. Participant identity was included as a random intercept. For these analyses, we included results only from analyses with the maximum number of database scans available for each study (this was 9 for MSC, 5 for NCANDA, and 1 for BNET).

Because we found that selecting 100-nodes from the Schaefer 1000 atlas resulted in lower identification performance than using all 1000 nodes, we believe that the number of nodes in the atlas is another key factor influencing how identifiable a scan is. Therefore, we fit a separate linear mixed effects model using only results from the Schaefer 100-node atlas and the average values (including number of correctly identified volumes, nodal tSNR, and nodal ReHo) from 100 iterations of down sampling the Schaefer 1000 atlas to 100 nodes. The formula of this linear mixed effects model was identical to the formula of those described in the prior paragraph – the only difference is that the observations from the Schaefer 200, Schaefer 500, and Schaefer 1000 node atlases were replaced with the average identification, tSNR, and ReHo values across the 100 down sampling iterations.

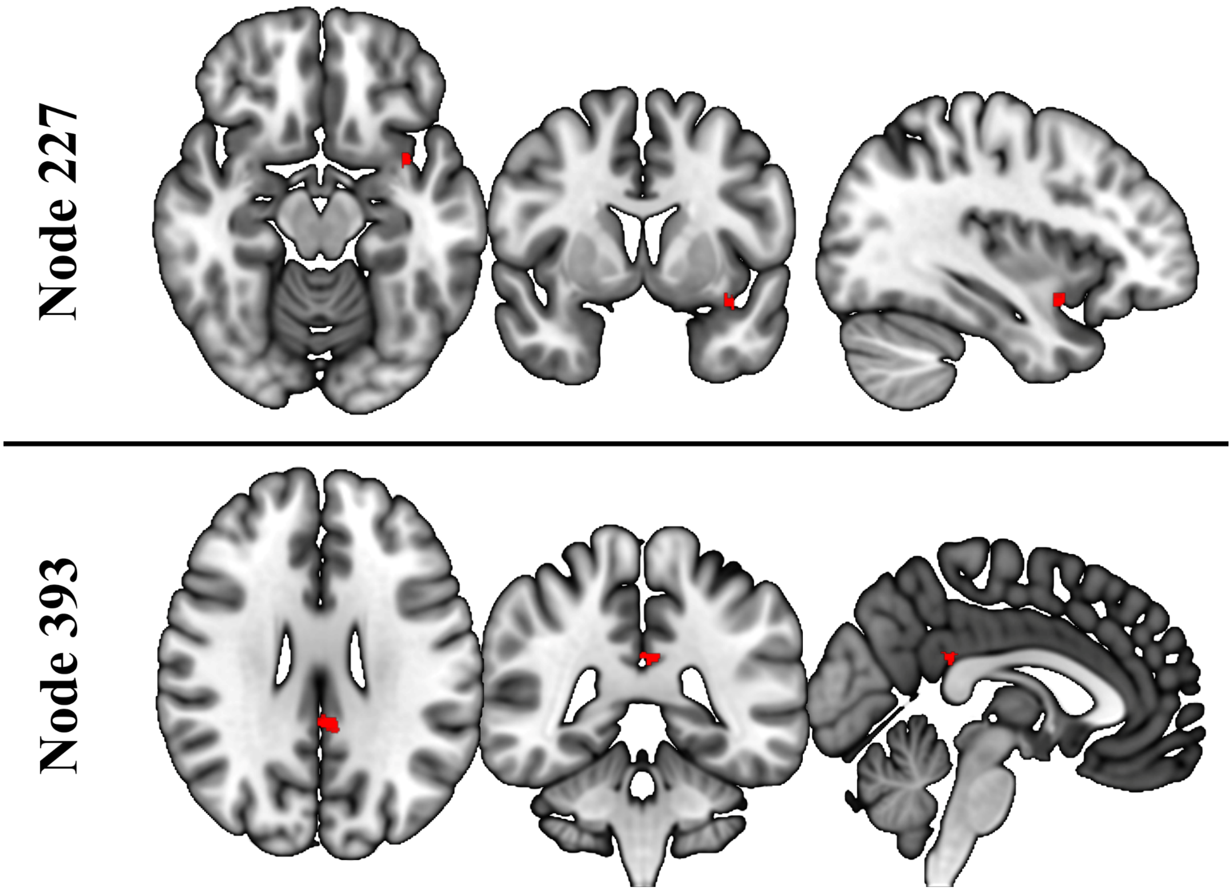

Supplementary Figure 1. Atlas nodes lost in reslicing of the Schaefer 1000-node (17 Subnetwork) atlas. Nodes are highlighted in red. Node 227 was lost in reslicing to 4x4x4mm (for MSC analyses) and 4x4x5 mm (for NCANDA analyses). Node 393 was retained in reslicing to 4x4x4mm but lost in reslicing to 4x4x5mm. Images are in radiological orientation.

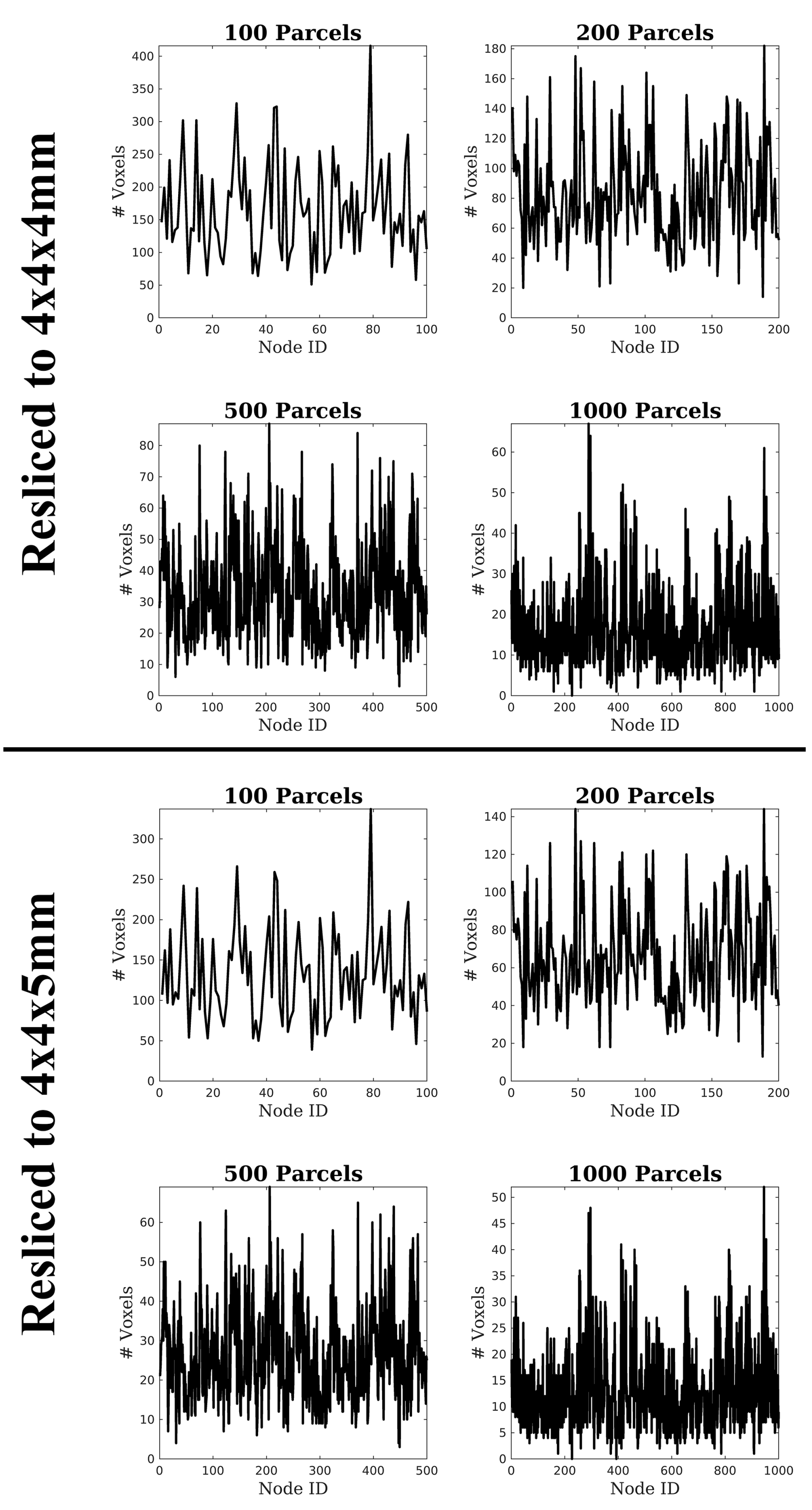

<u>Supplementary Figure 2.</u> The number of voxels in each node of the Schaefer atlases is shown for both the resliced 4x4x4mm atlas and the 4x4x5mm atlas.

Supplementary Table 1. MSC Individual Volume Identification Accuracy

	1 Database Session	5 Database Sessions	9 Database Sessions
Schaefer 100	41.4 ± 10.2	53.5 ± 8.7	57.4 ± 8.5
Schaefer 200	58.4 ± 12.5	74.1 ± 9.8	78.1 ± 9.2
Schaefer 500	77.7 ± 12.3	90.9 ± 7.2	93.3 ± 6.1
Schaefer 1000	86.8 ± 10.3	96.1 ± 4.9	97.4 ± 4.0

These values correspond to the data shown in Figure 6. Rows indicate different parcellations of the Schaefer atlas (see *Fingerprinting – Atlas Parcellation*) while columns indicate different numbers of database scans used (see *Fingerprinting – Number of Database Scans*). All values indicate percentage of volumes that correctly identified participants for the corresponding atlas and number of database sessions. Error (±) indicates standard deviation of individual scans.
MSC – Midnight Scan Club

Supplementary Table 2. MSC Full Scan Identification Accuracy

	1 Database Session		5 Database Sessions	9 Database Sessions
Schaefer 100	98.7		99.8	100
Schaefer 200	99.9		100	100
Schaefer 500	100		100	100
Schaefer 1000	100		100	100

These values correspond to the data shown in Figure 6. Rows indicate different parcellations of the Schaefer atlas (see *Fingerprinting – Atlas Parcellation*) while columns indicate different numbers of database scans used (see *Fingerprinting – Number of Database Scans*). All values indicate percentage of full scans that correctly identified participants across all iterations of different target scans and subjects.
MSC – Midnight Scan Club

Supplementary Table 3. NCANDA Individual Volume Identification Accuracy

	1 Database Session	3 Database Sessions	5 Database Sessions
Schaefer 100	7.7 ± 5.6	10.4 ± 6.5	11.8 ± 7.1
Schaefer 200	14.2 ± 9.3	20.6 ± 11.0	24.0 ± 11.8
Schaefer 500	27.0 ± 15.0	39.9 ± 16.3	46.3 ± 16.8
Schaefer 1000	37.2 ± 18.1	54.1 ± 18.0	61.6 ± 17.5

These values correspond to the data shown in Figure 6. Rows indicate different parcellations of the Schaefer atlas (see *Fingerprinting – Atlas Parcellation*) while columns indicate different numbers of database scans used (see *Fingerprinting – Number of Database Scans*). All values indicate percentage of volumes that correctly identified participants for the corresponding atlas and number of database sessions. Error (±) indicates standard deviation of individual scans.

NCANDA – National Consortium on Alcohol and NeuroDevelopment in Adolescence.

Supplementary Table 4. NCANDA Full Scan Identification Accuracy

	1 Database Session	3 Database Sessions	5 Database Sessions
Schaefer 100	59.0	81.8	89.3
Schaefer 200	80.1	95.8	97.7
Schaefer 500	93.2	98.9	99.7
Schaefer 1000	95.7	99.6	99.8

These values correspond to the data shown in Figure 6. Rows indicate different parcellations of the Schaefer atlas (see *Fingerprinting – Atlas Parcellation*) while columns indicate different numbers of database scans used (see *Fingerprinting – Number of Database Scans*). All values indicate percentage of full scans that correctly identified participants across all iterations of different target scans and subjects.
NCANDA – National Consortium on Alcohol and NeuroDevelopment in Adolescence

Supplementary Table 5. BNET Individual Volume Identification Accuracy

	1 Database Session
Schaefer 100	6.8 ± 6.3
Schaefer 200	12.4 ± 10.4
Schaefer 500	22.4 ± 16.5
Schaefer 1000	31.1 ± 20.8

These values correspond to the data shown in Figure 6. Rows indicate different parcellations of the Schaefer atlas (see *Fingerprinting – Atlas Parcellation*) while columns indicate different numbers of database scans used (see *Fingerprinting – Number of Database Scans*). All values indicate percentage of volumes that correctly identified participants for the corresponding atlas and number of database sessions. Error (±) indicates standard deviation of individual scans.
BNET – Brain Networks and Mobility

Supplementary Table 6. BNET Full Scan Identification Accuracy

	1 Database Session
Schaefer 100	43.1
Schaefer 200	63.6
Schaefer 500	78.9
Schaefer 1000	85.0

These values correspond to the data shown in Figure 6. Rows indicate different parcellations of the Schaefer atlas (see *Fingerprinting – Atlas Parcellation*) while columns indicate different numbers of database scans used (see *Fingerprinting – Number of Database Scans*). All values

indicate percentage of full scans that correctly identified participants across all iterations of different target scans and subjects.
BNET – Brain Networks and Mobility

Supplementary Table 7. MSC Individual Volume Identification Accuracy – No Gray Matter Regression

	1 Database Session	5 Database Sessions	9 Database Sessions
Schaefer 100	42.1 ± 7.7	54.3 ± 9.0	58.1 ± 9.4
Schaefer 1000	86.8 ± 8.3	96.3 ± 4.9	97.5 ± 4.0

Rows indicate different parcellations of the Schaefer atlas (see *Fingerprinting – Atlas Parcellation*) while columns indicate different numbers of database scans used (see *Fingerprinting – Number of Database Scans*). All values indicate percentage of volumes that correctly identified participants for the corresponding atlas and number of database sessions. Error (±) indicates standard deviation of individual scans.
MSC – Midnight Scan Club

Supplementary Table 8. MSC Full Scan Identification Accuracy – No Gray Matter Regression

	1 Database Session	5 Database Sessions	9 Database Sessions
Schaefer 100	97.9	99.4	99.0
Schaefer 1000	99.9	100	100

Rows indicate different parcellations of the Schaefer atlas (see *Fingerprinting – Atlas Parcellation*) while columns indicate different numbers of database scans used (see *Fingerprinting – Number of Database Scans*). All values indicate percentage of full scans that correctly identified participants across all iterations of different target scans and subjects.
MSC – Midnight Scan Club

Supplementary Table 9. MSC Individual Volume Identification Accuracy – High/Low Motion

	High Motion	Low Motion
Schaefer 100	37.7	57.5
Schaefer 200	64.1	78.1
Schaefer 500	85.2	93.4
Schaefer 1000	88.8	97.4

Rows indicate different parcellations of the Schaefer atlas (see *Fingerprinting – Atlas Parcellation*) while columns indicate volumes with high motion versus low motion. Accuracy is based on fingerprinting analyses with 9 database scans. All values indicate percentage of full scans that correctly identified participants across all iterations of different target scans and subjects.
MSC – Midnight Scan Club

Supplementary Table 10. NCANDA Individual Volume Identification Accuracy – High/Low Motion

	High Motion	Low Motion
Schaefer 100	7.3	12.0
Schaefer 200	15.0	24.4
Schaefer 500	30.3	46.9
Schaefer 1000	42.8	62.3

Rows indicate different parcellations of the Schaefer atlas (see *Fingerprinting – Atlas Parcellation*) while columns indicate volumes with high motion versus low motion. Accuracy is based on fingerprinting analyses with 5 database scans. All values indicate percentage of full scans that correctly identified participants across all iterations of different target scans and subjects.

NCANDA – National Consortium on Alcohol and NeuroDevelopment in Adolescence

Supplementary Table 11. BNET Individual Volume Identification Accuracy – High/Low Motion

	High Motion	Low Motion
Schaefer 100	5.4	6.9
Schaefer 200	9.7	12.5
Schaefer 500	19.0	22.6
Schaefer 1000	25.0	31.5

Rows indicate different parcellations of the Schaefer atlas (see *Fingerprinting – Atlas Parcellation*) while columns indicate volumes with high motion versus low motion. Accuracy is based on fingerprinting analyses with 1 database scan. All values indicate percentage of full scans that correctly identified participants across all iterations of different target scans and subjects.

BNET – Brain Networks and Mobility

Supplementary Table 12. MSC Static Network Identification Accuracy

	1 Database Session	5 Database Sessions	9 Database Sessions
Schaefer 100	96.1	99.6	100
Schaefer 200	99.0	100	100
Schaefer 500	100	100	100
Schaefer 1000	99.9	100	100

Percentage of scans for which the individual was correctly identified based on their static network. Rows indicate different parcellations of the Schaefer atlas (see *Fingerprinting – Atlas Parcellation*) while columns indicate different numbers of database scans used (see *Fingerprinting – Number of Database Scans*). All values indicate percentage of full scans that correctly identified participants across all iterations of different target scans and subjects.

MSC – Midnight Scan Club

Supplementary Table 13. NCANDA Static Network Identification Accuracy

	1 Database Session	3 Database Sessions	5 Database Sessions
Schaefer 100	71.7	89.7	93.5
Schaefer 200	83.4	95.3	97.7
Schaefer 500	90.6	97.6	98.5
Schaefer 1000	92.4	98.7	99.3

Percentage of scans for which the individual was correctly identified based on their static network. Rows indicate different parcellations of the Schaefer atlas (see *Fingerprinting – Atlas Parcellation*) while columns indicate different numbers of database scans used (see *Fingerprinting – Number of Database Scans*). All values indicate percentage of full scans that correctly identified participants across all iterations of different target scans and subjects.
NCANDA – National Consortium on Alcohol and NeuroDevelopment in Adolescence

Supplementary Table 14. BNET Static Network Identification Accuracy

	1 Database Session
Schaefer 100	49.4
Schaefer 200	62.1
Schaefer 500	74.0
Schaefer 1000	77.7

Percentage of scans for which the individual was correctly identified based on their static network. Rows indicate different parcellations of the Schaefer atlas (see *Fingerprinting – Atlas Parcellation*). All values indicate percentage of full scans that correctly identified participants across all iterations of different target scans and subjects.
BNET – Brain Networks and Mobility

Supplementary Table 15. Midnight Scan Club Task Identification

	**Faces**	**Words**	**Rest**
**Within Subject**	58.4 ± 10.6	46.4 ± 10.3	60.3 ± 14.4
**Between Subjects**	49.9 ± 9.0	32.1 ± 6.4	51.4 ± 8.5

Values associated with the bar plots in Figure 7. All values indicate percentage of volumes that correctly identified participants for the corresponding atlas and number of database sessions. Error (±) indicates standard deviation of individual scans. By random chance, task identification for individual volumes would be expected to be correct 33.3% of the time.
